# The Importance of Self-Identities and Habitual Behavior for Eating More Plant Foods

**DOI:** 10.3390/nu16234063

**Published:** 2024-11-26

**Authors:** Marzena Jeżewska-Zychowicz, Marta Sajdakowska, Jerzy Gębski, Małgorzata Kosicka-Gębska, Krystyna Gutkowska

**Affiliations:** Institute of Human Nutrition Sciences, Warsaw University of Life Sciences (SGGW-WULS), Nowoursynowska 159 C, 02-776 Warsaw, Poland; marzena_jezewska_zychowicz@sggw.edu.pl (M.J.-Z.); jerzy_gebski@sggw.edu.pl (J.G.); malgorzata_kosicka_gebska@sggw.edu.pl (M.K.-G.); krystyna_gutkowska@sggw.edu.pl (K.G.)

**Keywords:** plant-based diet, habits, self-identity, transition

## Abstract

Objectives: This study aimed to identify the predictors of eating more plant-based foods and legumes in terms of previous changes in consumption, habitual frequency of consumption, intention to reduce the amount of meat consumed, and health and environmental identities. Methods: A cross-sectional survey was conducted between June and September 2023 in 881 Polish adults. Based on two self-descriptors, four groups of respondents were identified: no health identity and no environmental identity (nHI-nEI), health identity but no environmental identity (HI-nEI), no health identity but environmental identity (nHI-EI), and both health identity and environmental identity (HI-EI). A logistic regression analysis was used to verify associations between habitual eating of red meat, white meat, and legumes, changes in their intake over the past two years, familiarity with plant-based substitutes for animal products, health, and environmental identity, declared intentions to eat less meat, and the intentions to eat more plant-based food and eat more legumes next year. Results: Individuals were more likely to increase their consumption of plant foods (OR: 1.99, *p* = 0.001), including legumes (OR: 1.79, *p* = 0.045), when they represented the HI-EI group, had increased their consumption of legumes in the past two years (OR: 2.91, *p* = 0.009, and OR: 2.15, *p* = 0.017, respectively), declared an intention to reduce meat (OR: 8.02, *p* < 0.001, and OR: 12.08, *p* < 0.001, respectively), but also occasionally consumed plant-based meat substitutes (OR: 1.76, *p* = 0.002, and OR: 2.61, *p* < 0.001, respectively). However, individuals were more likely to increase their consumption of plant foods, but not legumes, if they currently consumed legumes frequently (OR: 1.36, *p* = 0.009, and OR: 1.22, *p* = 0.111, respectively) and had previously limited their consumption of red meat (OR: 2.40, *p* < 0.001, and OR: 1.09, *p* = 0.717, respectively). Conclusions: The habitual frequency of eating red and white meat did not predict the increased consumption of plant foods in the future. It is equally important to increase people’s awareness of the impact of the food they consume on health and the environment to support their health and pro-environmental motivation for food choices. Public health and sustainability campaigns should develop new methods to reach populations less willing to change.

## 1. Introduction

The discussion about the relationship between food production, healthy eating, and sustainable development has recently become increasingly animated. The considerations are complex because of the multifaceted nature of this relationship, including environmental and health impacts, cultural acceptance, differences in nutritional requirements across populations, and the effectiveness of long-term dietary changes [1]. To address both environmental impacts and health benefits, optimization modeling is increasingly being used to create optimal diets from the perspective of environmental and health outcomes [2,3]. In many populations, reducing the consumption of animal foods while increasing the consumption of plant foods is necessary for health reasons, as it may help to reduce the risk of certain diseases, i.e., type 2 diabetes, coronary heart disease, and cardiovascular disease [4,5,6], and prevent certain diseases by controlling glycemic levels, blood pressure, lowering blood cholesterol, etc. [7,8]. In addition to macronutrients such as proteins, plant foods also contain many carbohydrates with different roles in developing diverse gut flora and a lot of micronutrients such as vitamins, minerals, and bioactive compounds [9]. Many studies confirm that plant-based proteins can lack some essential amino acids [10,11,12,13]. However, some studies indicate that in some plant foods, their quantity is sufficient, e.g., in lupin and pigeon peas [14,15]. Thus, the diversity of plant foods and the characteristics of the proteins they contain do not warrant such a formulation. As a result, dietary guidelines recommend a plant-based diet with whole grains, vegetables, fruits, legumes, nuts, and unsaturated oils [15,16]. Environmental recommendations also stipulate a higher consumption of plant-based foods because they are less environmentally harmful. However, according to Chaudhary et al. [17], dietary changes towards the lower consumption of animal products and higher consumption of plant foods improved nutrient intakes and reduced greenhouse gas emissions (GHGEs) and freshwater use only in North America and Europe. In contrast, increasing GHGEs and freshwater use were necessary to achieve adequate nutrient intakes in other countries. This shows that dietary changes recommended for health and the environment without considering the broader context do not always lead to health and environmental benefits. Moreover, consuming more plant-based foods in highly developed countries appears to be a huge challenge for consumers [18,19]. A reluctance to change, and the difficulties associated with it, result from various reasons. They are the subject of many studies [20,21], in which the attention concerns, among others, motives of food choice, including those related to health, taste preferences, environment, animal protection, or price [22], but also barriers and facilitators of change towards a plant-based diet [23,24,25].

Previous studies indicated that health and environment were the most common reasons to consider adopting a plant-based diet, and they were positively associated with diet sustainability indicators [26,27,28]. Apart from the motives linked to a decision about food consumption [22,29,30], identity as a behavioral motivator should also be considered when behavioral change is expected [31]. Self-identity, especially health identity, has been considered in studies on predicting behavioral intentions and behaviors [32,33,34]. Self-identity is the label people use to describe themselves [34]. Thus, “environmental self-identity” can be defined as the degree to which people think their behaviors are environmentally friendly [35]. In turn, health identity concerns how individuals perceive themselves concerning a healthy lifestyle [33]. Previous research has confirmed the important role of health identity in motivating health literacy and self-efficacy, which in turn influences healthy behavioral intentions [33] and behaviors [36,37]. So far, self-identity has been primarily studied in Western cultures [31,38,39]. Moreover, self-identity was expressed in different ways, considering values, i.e., health identity [33], or behaviors, i.e., meat-eater identity [38,39].

It can be expected that the stronger environmental self-identity, the more it can encourage one’s environmental actions [35], and similarly, such a relationship occurs in the case of health identity and health-beneficial behaviors [36,37]. However, consumers sometimes have conflicting identities [40]. For example, when communicating one’s identity to others, an individual may buy certain products while actively avoiding others, depending on who the recipients of this communication are [41]. Moreover, to our knowledge, few studies still consider self-identity in consumer segmentation [42], which could reduce potential conflicts between different self-identities, including the possible conflict between environmental and health identity and its impact on the actions taken. Self-identity is a key driver of the behaviors performed to maintain particular roles that adhere to self-identity [32], which may indicate a strong connection between identities and habits. Habit as an action performed regularly as part of one’s routine is, in turn, a construct that helps to predict choices, such as food choices and dietary behaviors [42]. What is important is that habits may play an essential role in preventing individuals from changes, for example, from adopting a plant-based diet [43,44]. People who declared no changes in food consumption, which could be caused by habitual eating, were characterized by the most established consumption patterns and, at the same time, very rarely replaced meat with plant protein products [45].

To efficiently promote a dietary transition to a plant-based diet, a better understanding of what influences the changes in plant food consumption is required. The aim of the study was twofold: to identify homogenous clusters of respondents with similar self-perceptions in terms of health and environment, which allows us to explain how they can be linked to eating more plant food, and to identify other factors facilitating the eating of more plant foods including legumes, among which changes made in the past 2 years in the consumption of red meat, white meat, and legumes; habitual frequency of consumption of these foods; familiarity and consumption of plant-based meat substitutes; and an intention to eat less meat and more plant-based foods in the following year were included. 

This study contributes to the literature by examining whether health and environmental identities are appropriate for identifying consumer segments, which in turn, together with habitual behaviors, can predict future changes in plant food consumption.

## 2. Materials and Methods

### 2.1. Study Design and Sample Collection

By sending an online questionnaire, a cross-sectional survey was conducted between June and September 2023. The survey invitation explained the study’s aim and ensured data confidentiality and anonymity. In addition, the invitation outlined the criteria to be met to participate in the study. Age 18 and older, consumption of red and white meat, and consumption of red or white meat at least once a week were indicated as criteria to be met. Before filling out the questionnaire, the respondent agreed to participate in the study by marking one of two answers, i.e., “yes” or “no”. Only those who marked “yes” answered the questionnaire. The entire study sample included 881 Polish adults from all regions of the country. The professional research agency carried out the recruitment process for the study. The sample’s selection criteria considered the Polish population’s representativeness in terms of the province, and then the sampling was quota-based according to gender, education, and place of residence.

### 2.2. Questionnaire

The questionnaire consisted of four parts. The first part assessed the habitual frequency of consumption of three groups of products, i.e., red meat, white meat, and legumes, in the last 3 months, the changes made to it over the past 2 years, and intended plat-food consumption in the next year. Questions from the validated Beliefs and Eating Habits Questionnaire (KomPAN) [46,47] were used, and respondents reported their habitual frequency of eating these foods, choosing one of the following answers: 1—less than once a month; 2—1–3 times a month; 3—once a week; 4—a few times a week; 5—once a day; 6—a few times a day. Moreover, in the next two questions, respondents declared their intentions to eat more plant-based food and less meat next year. The questions were as follows: “Do you intend to eat more plant-based food next year?” (yes/no) and “Do you intend to eat less meat next year?” (yes/no). If the person answered “Yes”, they were asked to indicate whether they intended to increase their consumption of legumes (yes/no). Changes in red meat/white meat/legume intake over the 2 years preceding the study were examined using the question, “How do you assess the changes in the consumption of three food groups over the past 2 years?”. The nature of the changes was defined as 1—“I have not consumed these products”; 2—“Consumption has decreased”; 3—“Consumption has not changed”; and 4—“Consumption has increased”.

In the second part of the questionnaire, respondents assessed their familiarity with plant-based substitutes for animal products (PBSs); the question “Are you familiar with or consume any plant-based substitutes for animal products?” was used. The respondent selected one of the following answers: “I am not familiar with such products”; “I am familiar with such products, but I do not consume them”; “I consume such products occasionally (once a month or less)”; “I consume such products regularly”).

In the third part of the questionnaire, self-identity was measured. Two self-descriptors were used to measure self-identity [48]. Respondents characterized themselves by considering the meanings they ascribe to health and the environment by responding to two statements: “I consider myself to be a person who cares about health” and “I consider myself to be a person who is oriented towards protecting the environment.” Scores were rated on a 5-point scale: 1 (completely disagree), 2 (disagree), 3 (neither agree nor disagree), 4 (agree), and 5 (completely agree). Scores from 1 to 3 were defined as no health identity (nHI) and no environmental identity (nEI), while scores 4 and 5 were defined as health identity (HI) and environmental identity (EI). Then, four categories of respondents in terms of self-identity were identified: 1/no health identity and no environmental identity (nHI-nEI), health identity but no environmental identity (HI-nEI), no health identity but environmental identity (nHI-EI), and both health identity and environmental identity (HI-EI).

In the last part of the questionnaire, the socio-demographic characteristics, including age (in years), place of residence, education, and self-reported financial situation, were assessed.

### 2.3. Statistical Analysis

Descriptive statistics were employed to present the characteristics of the study sample. Data were presented as a sample percentage (%) for categorical data or mean value and standard deviation (SD) for continuous data. The normality of the distribution of continuous variables was assessed using the normality Kolmogorov–Smirnov test, Lilliefors test, and normal probability plot. The Chi-square test for independence and Student’s *t*-test were employed to assess whether there were differences between subgroups. A statistical significance level of *p* < 0.05 was established.

A logistic regression analysis was used to verify associations between habitual eating (frequency of eating red meat, white meat, and legumes), changes in red meat/white meat/legumes intake over the past 2 years, familiarity with plant-based substitutes for animal products, health, and environmental identity, declared intentions to eat less meat (independent variables), and the intentions to eat more plant-based food and eat more legumes next year (dependent variables). Odds ratios (ORs) represented the probability of belonging to a group declaring an intention to eat more plant-based food (Model 1) and more legumes (Model 2) next year. The reference group (OR = 1.00) included those who did not express an intention. Wald’s test was employed to evaluate the significance of the odds ratios.

The statistical analysis was performed using IBM SPSS Statistics for Windows, version 29.0 (IBM Corp., Armonk, NY, USA).

## 3. Results

### 3.1. Description of the Study Sample

Table 1 presents the characteristics of the study sample. The sample comprised 881 adults, with 50.9% men and 49.1% women. The participants were 18–83 years old; the mean age was 45.7 years (standard deviation: 15.7). More than half of the respondents declared that they cared about their health (HI: 54.5%) and were environmentally oriented (EI: 57.7%).

### 3.2. Characteristics of Categories Identified by Health and Environmental Identities

The study group mainly consisted of people who were oriented towards both health and the environment (HI-EI—45.3%), followed by those who were neither oriented towards health nor the environment (nHI-nEI—23.3%). People who cared about their health but were not oriented towards the environment constituted 19.1% of the respondents (HI-nEI). In comparison, the lowest number of respondents did not care about their health but were oriented towards the environment (nHI-EI—12.3%).

The characteristics of the groups distinguished based on health and environmental identities are presented in Table 2. 

Among those who perceived themselves as both health-unconscious and not environmentally oriented (nHI-nEI), there were more men (56.1%) than women (43.9%), more people aged 18–24 (16.1%), and fewer people aged 65 and over (5.4%) than in the entire study group (10.0% and 12.4%, respectively). The group of people who cared about their health but were not environmentally oriented (HI-nEI) included mostly people aged 25–34 (28.0%) and fewer people aged 55–64 (14.3%). In the group of people who did not care about their health but were oriented towards the environment (nHI-EI), there were more men (59.3%) than women (40.7%), more people aged 55–64 (26.9%), and fewer people aged 25–34 (14.8%) compared to the entire study group (22.1% and 19.3%, respectively). In turn, in the group of people who cared about their health and were environmentally oriented (HI-EI), there were more women (54.2%) than men (45.8%), more people aged 55–64 (25.4%) and 65 and over (16.3%), and fewer people aged 18–24 (6.8%) compared to the entire study group (22.1%, 12.4%, and 10.0%, respectively) (Table 2). The remaining variables, i.e., education and place of residence, did not differentiate the subgroups identified based on self-identities.

### 3.3. Health Identity, Environmental Identity, and Consumption of Meat and Plant Food

There were no significant differences in the frequency of red meat consumption (mean = 3.16; SD = 0.98; median = 3; mode = 4), white meat (3.52; 0.89; 4; 4, respectively), and legumes (2.68; 0.96; 3; 2, respectively) between the groups identified based on health and environmental identity. However, these groups differed regarding changes in red meat and legume consumption over the two years preceding the study. Moreover, the groups were also different regarding the intention to eat more plant food and legumes as well as the intention to eat less meat food and more legumes (Table 3). 

Regarding the familiarity with and consumption of plant-based substitutes less environmentally oriented (EI) people, both health-conscious (HI) and non-health-conscious (nHI), were not familiar with plant-based meat substitutes (26.8% and 27.8%, respectively). In the nHI-EI group and in the HI-EI group, more people occasionally consumed plant-based substitutes (44.4% and 36.7%) (Table 3).

### 3.4. Predictors of Eating More Plant-Based Foods, Including Legumes

The likelihood of having an intention to eat more plant-based foods increased with an increase in the habitual frequency of eating legumes (by 36%), a decrease in intake of red meat over the previous two years (by 146%), an increase in intake of legumes over previous two years (by 191%), eating plant substitutes occasionally (by 76%), and perceiving oneself as both health- and environment-oriented person (HI-EI—by 99%). Most importantly, an 8-fold increase in the likelihood of such declarations was observed when the intention to eat less meat was declared (Table 4).

The likelihood of having an intention to eat more legumes increased with an increase in intake of legumes over the two years preceding the study (by 115%), eating plant substitutes occasionally (by 161%), eating plant substitutes regularly (by 536%), and perceiving oneself as both health- and environment-oriented person (HI-EI by 79%). The likelihood of making such a declaration increased approximately 13-fold when the intention to eat less meat was declared and 18-fold when the intention to eat more plant-based foods was declared (Table 4).

## 4. Discussion

The study aimed to determine if and how self-identities and habitual eating can explain future plant-food consumption. It was found that self-perceptions of being health-conscious and environmentally oriented at the same time (HI-EI), in addition to increased consumption of legumes in the previous two years, a declaration to reduce meat consumption, but also occasional consumption of plant-based meat substitutes increased the likelihood of increased consumption of plant foods in the future, including legumes. In contrast, the odds of increasing the intake of plant foods but not legumes increased when the habitual frequency of legume consumption was increased, and red meat consumption was already reduced during the 2 years preceding the study. It also found that habitual frequency of meat consumption was not a predictor of increased future consumption of plant foods. In contrast, the habitual frequency of legume consumption increased the odds of consuming more plant foods but not legumes in the future. 

Our results are generally in line with previous ones, which indicate an increase in the consumption of plant foods overall, including legumes [49,50], especially among environmentally conscious people [51,52]. This relationship was confirmed only in individuals who simultaneously represented a health and environmental identity (HI-EI) (Table 4). The lack of a relationship when individuals were environmentally oriented but health-unaware (nHI-EI) indicates that examining the sum of the effects of two factors, i.e., environmental self-assessment and health self-assessment, reveals differences in the influence of the environmental factor on the declared intentions to consume plant-based food. As far as we know, similar studies, i.e., on the relationship between value-based identity (health, environment) and dietary behaviors, have not yet been conducted, making it difficult to discuss the results obtained. As a result, the study showed the usefulness of the value-defined identity construct in explaining plant-based food consumption. Previous studies have already demonstrated the importance of self-identity in guiding dietary choices, e.g., the association between diet-related identity and diet quality [53], social identity and meat avoidance [54], and social identity and meat-free meal choices [55], but the links between self-identity and the decision to adopt a plant-based diet are still under-researched [55,56]. Thus, our results contribute to a better understanding of the relationship between value-based self-identity and plant-based food consumption and offer insights for dietitians, marketers, and policymakers to encourage a more sustainable diet.

Reductions in red meat consumption for environmental reasons were shown in environmental identity groups (EI), regardless of their health identity (HI) (Table 3). This may indicate that changes in red meat consumption are primarily driven by environmental concerns rather than by concerns for one’s health. Thus, several studies confirmed that ‘meat reducers’ reported environmental concerns as a reason for making efforts to reduce meat consumption [29,57,58,59]. However, studies indicate that environmental motives were not revealed as motives for reducing meat consumption [57,60] or were of moderate importance [61]. Some inconsistencies in the available results may be explained by the importance of many other factors determining food choice (i.e., health problems, social environment, nutritional education) but also by existing barriers (i.e., convenience, selfish and altruistic motives, price) limiting food behavior focused on sustainability [62]. Health issues are critical for consumers when they change their consumption patterns to include less or no meat [22,63]. However, the results of this study showed that self-perception, considering health and environment together, is a better predictor of increased consumption of plant-based foods than either of them taken separately.

Health and environmental identities did not determine the habitual frequency of eating white and red meat and legumes, which is difficult to explain. Such differences might have been expected only in red meat [58], treated as an ecological but also a health risk, especially in countries where its consumption is high [64,65,66,67], such as Poland [68]. Previous studies showed that health and environmental motives were positively associated with diet sustainability indicators [26,28], which suggested that differences would emerge when comparing “non-health and non-environment oriented” and “both health and environment-oriented” respondents. Although previous research confirmed the importance of health and environmental motives in determining behavioral intentions [33] and behaviors [36,37], it has also been shown that there is a gap between motives and actual sustainable consumption [69]. The lack of differences in the habitual frequency of eating red meat, white meat, and legumes between the groups separated by identities may indicate a weak relationship between this behavioral indicator and identity. In contrast, variation by self-identity was more related to changes in food consumption behavior, both made in the past and planned for the future.

This study’s results show that the frequency of legume consumption was a predictor of eating more plant foods, while it was not a predictor of eating more legumes (Table 4). It can, therefore, be assumed that the habitual frequency of eating legumes was satisfactory for the respondents and thus a barrier to eating them more often [43]. The literature regarding the importance of habit as a barrier to adopting PBDs is scarce. However, a Polish study showed that habits are the main barrier to consuming legumes [44]. Furthermore, not all plant-based foods, including legumes, are socially desirable in many developed countries [70]. For instance, in Europe, the average consumption of legumes is estimated at 7 g per capita per day, which is very low [70]. Thus, increasing legume intake remains challenging for the transition towards sustainable consumption. In contrast, it has been found that the frequent consumption of legumes can increase the intake of plant foods in general, which can help diversify the diet by increasing the proportion of plant foods [71] and indirectly reducing meat consumption.

The groups identified based on health and environmental identity were differentiated by gender and age (Table 2)—more women than men declared health and environmental identity (HI-EI). Findings from earlier studies confirm both women’s greater pro-environmental motivation [70], their greater health motivation [30,72,73,74] as well as differences in behaviors; for example, women are more likely than men to change their current dietary patterns, including increasing the amount of plant-based foods in their diets [45,75] and replacing meat dishes with those prepared with legumes [76]. 

Other studies confirm the results obtained in this study in the older age group regarding health values. Older adults demonstrate higher health motivation [71,72,73] and are more interested in healthy eating than younger individuals [77,78]. Some studies have shown that environmental concern and the commitment to pro-environmental activities are higher in older versus younger generations [79]. Nevertheless, other studies show that younger adults are characterized by higher environmental motivations [71,80], a higher awareness of the environmental impacts of food production [81], and a higher perception of environmental protection as a factor in reducing meat consumption [30]. It is stressed, however, that young adults also emphasized convenience and mood control motives more than middle-aged and older adults [73]. Previous findings suggest that each generation prefers environmentally friendly behavior [82], which can be readily observed in the family environment [83]. Because younger people are more likely to be affected by their environment in the future [84], they are probably more likely to be interested in its protection and more motivated to change their behavior. Nevertheless, the declared low interest in one’s health and limited orientation towards the environment among young adults indicates the need for further research in this age group to clarify the relationship between self-identity, motivation, and eating behavior and its changes. 

The absence of differences between identity groups after considering education is surprising, as other studies have shown that those with higher education prioritized health motives [30,85]. Moreover, a higher level of education was associated with health consciousness and awareness of the environmental impacts of food production [81]. However, in the literature, there are also studies with inconclusive results on the relationship between education and pro-environmental attitudes (e.g., [86]). Similarly, no differences in pro-environmental attitudes depending on education level were shown in another Polish study, and explained by the low tolerance of environmental issues among educated and young people despite their knowledge of the environment [87], which may also explain the previously discussed results obtained in the young adult group.

## 5. Strengths and Limitations

Our study brings new insights into the health and environmental motives for eating behaviors associated with consuming plant-based foods using the construct of self-identity. The strength of the study is a representative sample of the Polish population in terms of the province and the quota character of the sample regarding gender, education, and place of residence, which gives a significant potential for both scientific and practical application in developing strategies aimed at promoting more healthy sustainable consumption. This methodological approach can be applied to different populations, allowing comparisons under various cultural conditions, which are essential in determining the consumption of plant-based foods.

Besides the strengths mentioned above, this study has some limitations related to the data, which may contribute to some bias. Firstly, the study relied on self-reported information, which pose limitations such as reporting bias, recall bias, and difficulty in assessing, for example, changes made over the previous two years. We used only self-report measures of identity because we were interested in consciously accessible self-perceptions. However, future work examining self-identity should also include indicators that may lie outside people’s awareness and behavioral variables that are complementary to indicators describing an individual’s identity. Secondly, the study was conducted in one country, and as such, future studies should be expanded to other populations to learn about differences and similarities resulting from sociocultural backgrounds. Thirdly, this study focused on the relationship between the two motives, i.e., health and environment, and their total impact on the intention to consume more plant-based foods. The obtained results are promising. However, we suggest that in future studies, other motives should be considered as determinants of self-identity, e.g., attachment to tradition or religious beliefs. Although the most common reasons for adopting a plant-based diet in Western societies are health and environmental reasons, some people may have more specific reasons, such as religious beliefs or taste-related preferences, which justifies broadening the scope of the study. Incorporating cultural nuances and targeting more values can improve the generalizability and effectiveness of interventions promoting sustainable food consumption across cultures [88]. The study did not take into account existing barriers to the consumption of plant-based foods, including, among others, concerns about nutritional deficiencies (such as B12, iron) [29,89], food neophobia, and lack of information [23,25], which may modify the obtained results concerning primarily the relationship between self-identity and the consumption of plant-based foods. In addition, the study used a general construct, plant-based foods, which limits the applicability of the results to specific plant-based food groups. On the other hand, the additional inclusion of only one specific food group, such as legumes, may have focused the attention of some respondents on this food group when answering about plant-based foods.

## 6. Implications

Self-identities and habitual behaviors may influence future plant-based food consumption. The complexity of this relationship requires consideration when planning interventions to exploit their complementarity or limit the intervention to the decisive element. Past changes as predictors of future changes suggest developing recommendations for overcoming habits as a stabilizing factor in consumption, which may require modifying the environment to make plant foods more accessible. This could reduce physical and economic constraints and ensure that motivations related to health and environmental identity increase the chances of changing current consumption patterns. Furthermore, education through mass media campaigns could help raise the awareness of plant-based diets and promote social change. 

## 7. Conclusions

The findings of the study showed that an increase in the consumption of plant foods, including legumes, was more likely when both health identity and environmental identity characterized a person, and when they had increased the consumption of legumes in the previous two years, had the intention to eat less meat, but also occasionally ate plant-based meat substitutes. In addition, the chances of increasing the consumption of plant foods increased when legumes were frequently consumed, and the consumption of red meat had been previously limited. However, it turned out that the habitual frequency of meat consumption did not predict an increase in plant food intake in the future. In contrast, the habitual frequency of legume consumption increased only the chances of greater consumption of plant foods, not legumes.

Based on the results obtained, it is equally important to increase people’s awareness of the impact of the food they consume on health and the environment to support their health and environmental motivation for food choices. Public health and sustainability campaigns should develop new methods of incorporating health and environmental issues together to reach populations less willing to change. Differences between demographic groups, especially gender, highlight potential targets for future campaigns promoting a healthy, sustainable diet. Policies addressing gender differences in the future should aim to facilitate more nutritious and more sustainable choices and behaviors among male consumers.

## Figures and Tables

**Table 1:** Characteristics of the study sample (N = 881).

Socio-Demographic Characteristics	Total Sample (N * = 881) % (N)
Gender	
Male	50.9 (448)
Female	49.1 (433)
Age	
18–24 years old	10.0 (88)
25–34 years old	19.3 (170)
35–44 years old	20.1 (177)
45–54 years old	16.1 (142)
55–64 years old	22.1 (195)
65 years and above	12.4 (109)
Education	
Primary	9.4 (83)
Vocational	17.6 (155)
Secondary	40.4 (356)
Higher	32.6 (287)
Place of residence	
A village	37.9 (334)
A town with fewer than 20,000 inhabitants	13.6 (119)
A city with 20,000–100,000 inhabitants	17.9 (158)
A city with over 100,000 inhabitants	30.6 (270)
Self-reported financial situation	
There is enough for everything without much saving	17.1 (150)
We live frugally, and we have enough means for everything	37.9 (334)
We live very frugally to save for major purchases	27.1 (239)
There is only enough money for the cheapest food and clothing	8.4 (74)
There is only enough money for the cheapest food, not enough for clothing	3.9 (34)
There is not enough money even for the cheapest food and clothing	1.2 (11)
It is difficult to say	4.4 (39)
Presence of children	
With children	42.3 (373)
Without children	57.7 (508)
Self-identity	
Health identity (HI) **	54.5 (568)
No health identity (nHI) **	45.5 (313)
Environmental identity (EI) ***	57.7 (508)
No environmental identity (nEI) ***	42.3 (373)

* N—number of participants; ** HI—answers 4–5 for self-perception as a health-caring person; nHI—answers 1–3 for self-perception as a health-caring person (1—completely disagree; 5—completely agree); *** EI—answers 4–5 for self-perception as an environmentally oriented person; nEI—answers 1–3 for self-perception as an environmentally oriented person (1—completely disagree; 5—completely agree).

**Table 2:** Sociodemographic characteristics of the subgroups identified by health and environmental identities (N = 881).

	Self-Identity with Regard to Health and Environment				Total Sample
	No Health Identity and No Environmental Identity (nHI-nEI)	Health Identity but No Environmental Identity (HI-nEI)	No Health Identity but Environmental Identity (nHI-EI)	Both Health Identity and Environmental Identity (HI-EI)	
Number of respondents	205	168	108	400	881
Gender (*p* = 0.023)					
Male	56.1 (115)	51.2 (86)	59.3 (64)	45.8 (183)	50.9 (448)
Female	43.9 (90)	48.8 (82)	40.7 (44)	54.2 (217)	49.1 (433)
Age (*p* < 0.001)					
18–24	16.1 (33)	10.1 (17)	10.2 (11)	6.8 (27)	10.0 (88)
25–34	20.0 (41)	28.0 (36)	14.8 (16)	16.5 (66)	19.3 (170)
35–44	22.9 (47)	21.4 (36)	20.4 (22)	18.0 (72)	20.1 (177)
45–54	16.1 (33)	14.3 (24)	15.7 (17)	17.0 (68)	16.1 (142)
55–64	19.5 (40)	14.3 (20)	26.9 (13)	25.4 (65)	22.1 (109)
65 and above	5.4 (11)	11.9 (20)	12.0 (13)	16.3 (65)	12.4 (109)

**Table 3:** Changes in food intake over the two years preceding the study, intention to eat more plant food and less meat, familiarity, and consumption of plant-based substitutes in the subgroups identified by health and environment identities (N = 881).

	Self-Identity About Health and the Environment				Total Sample
	No Health Identity and No Environmental Identity (nHI-nEI)	Health Identity but No Environmental Identity (HI-nEI)	No Health Identity but Environmental Identity (nHI-EI)	Both Health Identity and Environmental Identity (HI-EI)	
	N * = 205	N = 168	N = 108	N = 400	N = 881
Changes in red meat intake over the past two years (*p* = 0.020)					
Intake decreased	34.6 ^a^ (71)	39.3 ^a,b^ (66)	48.2 ^a,b^ (52)	48.8 ^b^ (195)	43.6 (384)
No changes	60.0 ^a^ (123)	57.7 ^a,b^ (97)	47.2 ^a,b^ (51)	46.4 ^b^ (186)	51.9 (457)
Intake increased	5.4 ^a^ (11)	3.0 ^a^ (5)	4.6 ^a^ (5)	4.8 ^a^ (19)	4.5 (40)
Changes in white meat intake over the past two years (*p* = 0.499)					
Intake decreased	18.5 (38)	23.8 (40)	25.0 (27)	23.5 (94)	22.6 (199)
No changes	71.3 (146)	64.3 (108)	63.9 (69)	62.3 (249)	64.9 (572)
Intake increased	10.2 (21)	11.9 (20)	11.1 (12)	14.2 (57)	12.5 (110)
Changes in legume intake over the past two years (*p* = 0.022)					
No intake	5.9 ^a^ (12)	4.8 ^a^ (8)	7.4 ^a^ (8)	3.5 ^a^ (14)	4.8 (42)
Intake decreased	22.9 ^a^ (47)	23.2 ^a^ (39)	17.5 ^a^ (19)	17.0 ^a^ (68)	19.6 (173)
Without any changes	64.4 ^a^ (132)	61.3 ^a^ (103)	59.3 ^a^ (64)	63.2 ^a^ (253)	62.7 (552)
Intake increased	6.8 ^a^ (14)	10.7 ^a,b^ (18)	15.7 ^a,b^ (17)	16.3 ^b^ (65)	12.9 (114)
Intention to eat more plant food and more legumes (*p* < 0.001)					
No intention	53.1 ^a^ (109)	48.2 ^a,b^ (81)	35.2 ^b,c^ (38)	28.0 ^c^ (112)	38.6 (340)
More plant food but not of legumes	41.0 ^a^ (84)	42.9 ^a,b^ (84)	56.5 ^a,b^ (61)	54.7 ^b^ (219)	49.5 (436)
More plant food, including more legumes	5.9 ^a^ (12)	8.9 ^a,b^ (15)	8.3 ^a,b^ (9)	17.3 ^b^ (69)	11.9 (105)
Intention to eat less meat food and more legumes (*p* < 0.001)					
No intention	68.8 ^a,b^ (141)	71.4 ^b^ (120)	53.7 ^a,c^ (58)	47.1 ^c^ (191)	57.8 (510)
Less meat but no more legumes	18.0 ^a^ (37)	13.1 ^a^ (22)	18.5 ^a^ (20)	18.3 ^a^ (73)	17.3 (152)
Less meat and more legumes	13.2 ^a^ (27)	15.5 ^a,b^ (26)	27.8 ^b,c^ (30)	34.0 ^c^ (136)	24.9 (219)
Familiarity with and consumption of plant-based substitutes (*p* = 0.002)					
I am not familiar with	40.0 ^a^ (82)	38.1 ^a^ (64)	27.8 ^a,b^ (30)	26.8 ^b^ (107)	32.2 (283)
I am familiar with, but I have never eaten	28.4 ^a^ (58)	29.2 ^a^ (49)	24.1 ^a^ (26)	28.7 ^a^ (115)	28.1 (248)
I eat occasionally (once a month or less often)	28.7 ^a^ (59)	28.5 ^a^ (48)	44.4 ^b^ (48)	36.7 ^a,b^ (147)	34.3 (302)
I eat regularly	2.9 ^a^ (6)	4.2 ^a^ (7)	3.7 ^a^ (4)	7.8 ^a^ (31)	5.4 (48)

* N—number of respondents. ^a, b, c^—values in the same line with the same letter are not significantly different; Chi-square test, *p* < 0.05.

**Table 4:** Predictive models for intention to eat more plant food next year and intention to eat more legumes next year.

Variables	Intention to Eat More Plant Food Next Year (Model 1)					Intention to Eat More Legumes Next Year (Model 2)				
	β	e^β^	95% CI *		*p*-Value *	Β	e^β^	95% CI		*p*-Value
Frequency of eating red meat	0.009	1.01	0.84	1.21	0.919	0.011	1.01	0.80	1.27	0.926
Frequency of eating white meat	−0.069	0.93	0.77	1.14	0.494	0.047	1.05	0.81	1.36	0.723
Frequency of eating legumes	0.308	1.36	1.12	1.66	0.002	0.199	1.22	0.96	1.56	0.111
Intake of red meat decreased (ref. without changes in red meat intake)	0.876	2.40	1.60	3.60	<0.001	0.090	1.09	0.67	1.78	0.717
Intake of red meat increased (ref. without changes in red meat intake)	0.692	2.00	0.85	4.72	0.114	−0.755	0.47	0.16	1.41	0.179
Intake of white meat decreased (ref. without changes in white meat intake)	−0.378	0.68	0.41	1.14	0.143	0.120	1.13	0.66	1.92	0.658
Intake of white meat increased (ref. without changes in white meat intake)	0.388	1.47	0.84	2.59	0.178	0.203	1.23	0.64	2.34	0.537
Intake of legumes decreased (ref. without changes in legume intake)	−0.177	0.84	0.55	1.29	0.418	−0.402	0.67	0.37	1.20	0.178
Intake of legumes increased (ref. without changes in legume intake)	1.069	2.91	1.31	6.50	0.009	0.764	2.15	1.15	4.02	0.017
I am familiar with, but I do not consume PBSs (ref. I am not familiar with)	0.262	1.30	0.86	1.97	0.215	0.232	1.26	0.70	2.26	0.435
I consume PBSs occasionally (ref. I am not familiar with)	0.564	1.76	1.16	2.66	0.007	0.959	2.61	1.53	4.45	<0.001
I consume PBSs regularly (ref. I am not familiar with it)	1.054	2.87	0.98	8.45	0.056	1.849	6.36	2.33	17.32	<0.001
Health identity and no environmental identity (HI-nEI) (ref. no health and no environmental identities: nHI-nEI)	0.283	1.33	0.82	2.16	0.255	0.443	1.56	0.74	3.27	0.242
No health identity but environmental identity (nHI-EI) (ref. no health and no environmental identities: nHI-nEI)	0.453	1.57	0.88	2.80	0.124	0.248	1.28	0.59	2.77	0.529
Both health and environmental identity (HI-EI) (ref. no health and no environmental identities: nHI-nEI)	0.691	1.99	1.31	3.03	0.001	0.580	1.79	1.00	3.20	0.049
Intention to eat less meat	2.082	8.02	5.32	12.09	<0.001	2.551	12.82	7.69	21.38	<0.001
Intention to eat more plant food						2.879	17.79	6.28	50.36	<0.001

* OR—point estimate (e^β^), 95% confidence intervals; significance level of the Wald’s test.

## Data Availability

The data are not publicly available because the data have not yet been made available in publicly available databases. However, the data presented in the study are available on request from the corresponding author.
